# Explanatory models of adult patients with type 2 diabetes mellitus from urban centers of central Ethiopia

**DOI:** 10.1186/s13104-016-2248-3

**Published:** 2016-09-13

**Authors:** Bruck M. Habte, Tedla Kebede, Teferi G. Fenta, Heather Boon

**Affiliations:** 1 School of Pharmacy, College of Health Sciences (CHS), Addis Ababa University (AAU), P. O. Box 1176, Addis Ababa, Ethiopia; 2 School of Medicine, CHS, AAU, P. O. Box 1176, Addis Ababa, Ethiopia; 3 Leslie Dan Faculty of Pharmacy, University of Toronto, 144 College Street (Room 514), Toronto, ON M5S 3M2 Canada

**Keywords:** Type 2 diabetes, Explanatory models, Kleinman’s model, Ethiopia, Qualitative research, Addis Ababa, Butajira

## Abstract

**Background:**

Type 2 diabetes, which is increasing as a public health problem in the low resource settings of Africa has been associated with the high prevalence of micro-vascular complications and increasing levels of macro-vascular complications. There is evidence from the developed world that understanding patient perceptions of chronic illness is important to design effective strategies for helping patients manage these conditions. This study utilized Kleinman’s model to explore the illness perceptions of type 2 diabetes patients attending treatment in Addis Ababa and Butajira (Ethiopia) and better understand how they manage their illness.

**Design:**

Qualitative interviews were conducted to elicit the explanatory models of purposively sampled type 2 diabetes patients attending treatment in three hospitals in central Ethiopia until saturation of key emerging themes was achieved. Analysis of interview transcripts was guided by Kleinman’s model.

**Results:**

A total of 39 participants, 24 from Addis Ababa and the rest from Butajira took part in the study. This study revealed that patients’ explanatory models were informed by both the traditional and biomedical models with emotional distress evident in some of the participants. The traditional model seemed to reflect the strong religious and cultural influences for the majority of study participants. The findings also revealed that symptoms played significant roles in how patients viewed their illness including assessment of its severity. Most were uncertain about the cause of their illness, with those expressing certainty citing factors over which they believed they had little or no control. This may have contributed to the perceptions about the use of religious healing and traditional medicines in a complementary or alternative manner to the biomedical regimen which could affect their adherence to recommended regimens and their health outcomes.

**Conclusion:**

This study suggests the need for a strong diabetes care program that is sensitive to patients’ experiences of their illness including emotional distress. Individuals providing the diabetes care should consider local and individual contexts and strive to make their approach patient-centered and engage active participation of patients. There appears to be a need for better training of health providers in different areas including health communications and the fundamentals of mental healthcare.

## Background

Non-communicable diseases such as diabetes mellitus are increasingly becoming major public health problems in low and middle income countries [1]. With regards to diabetes, estimates suggest that there are more than 415 million adults worldwide with diabetes, with this number expected to rise to 642 million by 2040 [2]. Approximately 4 out of 5 people with diabetes live in low- and middle-income countries with the number of people with type 2 diabetes mellitus increasing in every country. More than 321,000 deaths in the African region are estimated to be attributed to diabetes with 79 % of these deaths occurring in people below the age of 60, which is the highest proportion among the world’s seven regions. In Ethiopia, there are an estimated 1.3 million adult diabetes cases with a prevalence estimate of 2.9 % for the year 2015 in which 23,145 diabetes-related deaths were also reported [2].

Non-communicable diseases such as diabetes, cancer and stroke are now the leading cause of premature death among adults in Addis Ababa (Ethiopia) while the focus of the healthcare system is still geared toward addressing communicable diseases [3]. Studies done among patients with diabetes in different parts of the country have reported poor health outcomes including unacceptably high levels of blood glucose and blood pressure levels, micro- and macro-vascular diabetes-related complications, diabetic foot and other skin infections as well as hypercholesterolemia and hypertriglyceridemia [4].

Healthcare for chronic conditions such as diabetes requires the active involvement of patients given that they are the ones responsible for the bulk of self-management activities, be it medication-taking or other relevant lifestyle modifications. Different studies have reported how adherence to the recommended biomedical regimens and involvement of patients are affected by their perceptions about diabetes and its treatment [5]. Illness perceptions and adherence have been shown to be associated with patient outcomes [6]. Many of these studies have used theoretical models to guide the inquiry to elicit patient perceptions towards their illness and resulted in interventions aimed at impacting illness perceptions to improve adherence to recommended treatment regimens and improve patient outcomes [6].

Recent reports from hospital-based studies on the medication-taking behavior of patients with diabetes in southwestern Ethiopia have reported low levels of adherence (nearing 50 % in one of the studies) to recommended regimens [7, 8]. One of these studies reported that 33 % of the 267 participants had depressive symptoms which in turn was significantly associated with non-adherence to medication regimens. This in turn was associated to diabetes-related hospitalizations [8]. Studies of quantitative design give limited emphasis to patients’ perceptions, and there is dearth of literature that has explored patients’ explanatory models of their illness that could serve as basis for further interventions that incorporated individual and local contexts. The aim of this study was to conduct a qualitative, exploratory study to elicit the personal viewpoints or explanatory models of patients with type 2 diabetes that are attending treatment in Addis Ababa and Butajira.

### Theoretical model

Individuals have their own explanatory models about health and illness that could influence their health seeking behavior, adherence to treatment regimens and health outcomes. Understanding these explanatory models is therefore essential for the effective delivery of healthcare. These explanatory models appear to be informed by personal, family, social and cultural beliefs, occupation, religious affiliation, education and past experience with illness and healthcare among other things. Patients’ perceptions and popular explanations generally include similar issues as described in healthcare providers’ explanatory models which are: etiology; onset of symptoms; pathophysiology; course of illness (including type of sick role—acute, chronic, impaired—and severity of disorder); and treatment [9].

According to Kleinman’s work on explanatory models, patients and healthcare providers have different versions of the sickness condition–the illness aspect for the patient and the disease version for the provider. The illness problems for patients constitute the entire disorder including the difficulties of living with the sickness condition. In contrast, providers often disregard the illness problems and focus on the disease as the disorder. This difference in outlook is in part responsible for inadequate clinical care, patient and family dissatisfaction with professional care and patient non-adherence to recommended treatment regimen. It is highly recommended therefore for providers to understand patients’ explanatory models so as to be able to incorporate patients’ perceptions and concerns into clinical decision making. The critical points of difference between provider and patient perspectives, especially those that are believed to affect appropriate care, can be the focus for clear explanation, patient education and frank negotiation [9].

Kleinman’s model has been used as a framework in a variety of studies investigating patients’ viewpoints towards their illness and its influence on how they manage it. Most of these studies are from Western countries. The few studies done among ethnic minorities or immigrants in Western countries [10, 11] and non-western countries [12] have reported perceptions that combined the biomedical and cultural (folk) models to varying degrees. For example, a study done among Mexican–American patients with diabetes living along the US-Mexican border reported that their explanatory model was a synthesis of both Western biomedicine and traditional Mexican beliefs. This was especially evident in their expressions of their beliefs about the cause of type 2 diabetes and its treatment. With regards to the cause, many of the study participants believed that ‘susto’ (a fright or scare) was the cause of their illness while also citing that biomedical causes such as heredity, obesity, poor diet and lack of exercise may have played contributory roles [10].

A similar study to assess the perceptions towards diabetes of Hispanic migrant farm workers living in the US revealed combined biomedical and traditional belief systems. While some study participants did not know the cause of diabetes, others provided combined beliefs to describe the causes. With regards to the majority who were discussing biomedical factors as causes, most also reverted to a traditional belief model where life stressors were considered as precipitating factors to diabetes. The majority also upon diagnosis believed that diabetes was an acute illness that would be cured after completing the medication regimen. Another finding of this study was the self-report by the majority of developing depression following their diabetes diagnosis [11]. Such findings are indicative of the need to consider not only the biomedical model but also the traditional belief models and patient realities of living with diabetes to be able to effectively support them in its management. There is however dearth of similar literature dealing with such non-communicable diseases in African and especially in the Ethiopian setting that could better inform the management of patients with diabetes.

## Methods

Qualitative methods were selected as the most appropriate for the initial exploration of these intricate issues and to gain deeper understanding of patients’ explanatory models towards their illness. This study received ethical approval from the Institutional Review Board of the College of Health Sciences, Addis Ababa University (protocol number 036/13/PSP).

### Study settings

The study sites were 3 public hospitals in central Ethiopia. Tikur Anbessa Specialized Hospital (hereafter referred to as Tikur Anbessa) and Yekatit 12 Hospital (hereafter referred to as Yekatit 12) serve a high number of patients with type 2 diabetes in Addis Ababa (the capital city of Ethiopia). In contrast, Butajira Hospital (hereafter referred to as Butajira) is the only public hospital in Butajira and was included to investigate the perspectives of patients living in the peri-urban part of central Ethiopia. A brief description of the two urban settings is given in Table 1.

**Table 1:** Description of study settings

	Addis Ababa	Butajira
Significance	Largest urban center in Ethiopia	Home to the demographic surveillance site of AAU
Population [35]	3.2 million	63 thousand
Ethnic groups [36]	Amhara (47 %), Guragie (16.3 %), Oromo (19.5 %) and Tigrie (6.2 %)	Guragie (82 %)
Religions [36]	Orthodox Christianity (74.7 %), Islam (16.2 %) and Protestant Christianity (7.8 %)	Islam (51.3 %), Orthodox Christianity (39.6 %) and Protestant Christianity (8.7 %)
Literacy [36]	85.3 %	37.9 %

Tikur Anbessa is the highest referral university hospital in the country. Patients diagnosed with diabetes are seen in the Diabetes Center which during the study period was run by 3 endocrinologists and 2 endocrinology fellows who work as consultants on a rotating basis, up to 6 Internal Medicine residents assigned to take the primary role in managing the patients doing their month long attachments, 6 nurses and 1 recently recruited pharmacist.

Yekatit 12 is a general hospital which has recently started training medical doctors is managed by the city administration. The services provided for diabetic patients were primarily in the general outpatient department run by 4 general practitioners while problematic cases were referred to the medical referral clinic run by internists on a rotating basis. In both Tikur Anbessa and Yekatit 12, patients are ‘randomly’ assigned to a doctor each time they visit.

Butajira is a general hospital that serves diabetic patients at a separate medical clinic run by a general practitioner and a nurse. In this case, diabetic patients would meet the same doctor when they come every month for at least a 6 month period.

### Study participants recruitment

The study participants were purposively selected patients with diabetes attending their treatment in the selected hospitals during the study period. The inclusion criteria included age of 18 years and older, being on anti-diabetic medications for minimum of 1 year and having no known or overt psychiatric problems while being a healthcare professional was an exclusion criterion. Apart from these criteria, patients were purposively selected to include a wide variation in terms of socio-demographics (sex, age, marital status, educational level, religious affiliations, employment status, place of residence), income level and years since diagnosis.

### Interview methods

Individual in-depth interviews (completed in Amharic) were conducted by the first author from December 2013 to March 2014. The interviews which had a median duration of 49 min (and ranged from 30 to 120 min) were audio recorded with participants’ consent. The set of questions recommended by Kleinman et al. [9] were largely used to frame the interview guide (as shown in the ‘‘Appendix’’) with minor modifications made as per related literature [9]. Patients were asked to discuss their explanatory model including about symptoms, course of their illness, problems caused by their diabetes, cause and treatment. The interview guide was translated to Amharic and back to English to check the consistency before using the Amharic version.

### Data analysis

The collected data were transcribed into MS Word by an experienced research assistant. The first author checked the quality of the transcripts by simultaneously listening to randomly selected audio recordings from each study site while reading the transcripts. The transcripts were then read repeatedly to ensure a good understanding before initially coding them according to the categories recommended by Kleinman’s model [9]. For some of those which did not fit with the model, open coding was done to accommodate them into separate categories and subcategories. The coding process in each site continued until all key themes were saturated, i.e. no new information was emerging [13]. While initial coding and categorization was done in Amharic, further analysis, and interpretation was carried out after translating key components of the transcripts relevant to the emerging themes into English. In this regard, BMH worked collaboratively with HB to analyze and interpret key findings until consensus was reached. NVivo 10 was utilized to manage the data.

## Results

Forty-five patients who met the eligibility criteria were identified during the study period. Of those, 6 did not participate either due to personal reasons such as being too busy or because of problems with telephone communication. A total of 39 study participants participated in the in-depth interview, of whom 24 were residents of Addis Ababa following treatment at Tikur Anbessa and Yekatit 12 and the rest of Butajira town or its environs. Table 2 provides a summary of the demographic characteristics of the study participants.

**Table 2:** Study participants’ characteristics (n = 39)

Description	Number of patients
*Sex*	
Female	19
Male	20
*Age (years)*	
30–39	2
40–49	8
50–59	14
60–69	10
>70	5
*Religion*	
Orthodox Christian	29
Muslim	8
Protestant	2
*Marital status*	
Married	27
Widowed	8
Divorced/single	4
*Educational status*	
Illiterate	6
Basic literacy (read and/or write)	10
Elementary complete	8
Secondary school complete	8
Post-secondary school education	7
*Occupation*	
Clerical work	7
Rents house	4
Small business	5
Farming	5
Pensioner	9
Unemployed	5
Others	4
*Diabetes duration (years)*	
1–5	10
6–10	14
11–15	7
16–20	4
21–25	4

Study participants’ explanatory models of illness are presented below, organized into six themes: labeling diabetes, symptoms, course of illness, impact of diabetes, cause and treatment. There were minimal differences in relation to the illness representations among the participants from the different study sites and thus the data were combined into a single set.

### Labeling diabetes

The Amharic term for diabetes is *‘ye sequar beshita’,* literally translated as ‘sugar disease’. ‘*Sequar’* (as it is called for short) has a negative and bitter connotation for many of the participants as depicted by emotion-laden expressions. Diabetes is considered by many to be a disease of the rich because they believe that diabetes comes to those who indulge in extravagances in their lifestyle especially with regards to diet or that the care required for managing the disease can only be afforded by the rich.

*They call it ‘sequar’ (literal translation is sugar) as if it were as nice; it is very bitter for those who tasted it (Female, 55* *years, diploma graduate, 9* *years with diabetes)*

*I thought that those who get diabetes were those who spend all their day eating fatty meat and drinking tej (a homemade wine made from honey). (Male, 70* *years, diploma graduate, 25* *years with diabetes)*

### Symptoms

The experience of symptoms was expressed in relation to learning of diabetes status and in assessing illness severity.

*Learning of diabetes status*: Participants described two primary routes to learning of their diabetes diagnosis: experience of different symptoms that ultimately led to their seeking care and chance diagnosis. Common symptoms such as increased thirst and urination frequency, weakness and loss of weight led most to seek healthcare. The vast majority (including those who had close family members with diabetes) did not relate the symptoms to diabetes. There were also a few who suspected that the symptoms could be indicative of HIV/AIDS, which was an important component of their reaction to the symptoms.

*For my case it suddenly started this time last year. I was very sick…. they took me to the hospital… When it started, I was unable to talk, extremely thirsty, couldn’t control my urine* - *the urge was constant and I couldn’t hold off (till I reached the toilet). (Male, 45* *years, high school, 2* *years with diabetes)*

*Initially, I used to urinate a lot; also drank water a lot… When my condition further worsened and I continued to lose weight and couldn’t even go to the toilet by myself, I wondered if I might have contracted AIDS. (Male, 57* *years, elementary school, 15* *years with diabetes)*

Some were diagnosed when they sought care for other conditions and since they did not identify diabetes-related symptoms it made it more difficult for them to accept the diagnosis of diabetes:

*When I went to be treated for another illness they told me I had diabetes. And I inquired, “What is diabetes? How does it work?”, as I didn’t know anything about it. … But I didn’t believe the laboratory technician and so went to a different place to get checked. (Male, 61* *years, elementary school, 15* *years with diabetes)*

Strong emotions such as being startled, upset and hopelessness were expressed upon being informed of diabetes diagnosis:

*I was very startled (when I was told of my diabetes). I didn’t think I would walk. (Female, 73* *years, low education, 20* *years with diabetes)*

*Symptoms used to assess illness severity*: Symptoms, or their absence thereof, and the carrying of sweets (to protect against hypoglycemia) were used to assess illness severity.

*My type doesn’t have any symptoms, none of the illness indication. But the other type has excessive urination; I think it also has low sugar levels. The medicines are the same but (those with) the other type also take sweets when it goes down. I think that one is worse. (Female, 50* *years, diploma graduate, 14* *years with diabetes)*

### Course of illness

*Perceived onset*: Participants often identified specific incidents they considered the starting point of the illness. Many linked the beginning of their illness to an episode of sudden rage or similar emotional moments. There were a few however who surmised that the illness may have been with them without being detected for a quite a while.

*When it started it did so with anger. Incidentally I was on leave and they shifted me from my job position which my friends told me. Soon after, I had a burning feeling here on my side. I also started drinking ten bottles of water every day (Female, 54* *years, elementary school, 2* *years with diabetes)*

*When I went back to the hospital where I had the operation they asked, ‘Where did the diabetes come from? It was not there when you had the operation. So did you get angry or get stressed? To which I responded, ‘Yes I got angry’. (Female, 60* *years, low education, 8* *years with diabetes)*

*Understanding the chronic nature*: Many participants expected diabetes to be an acute condition that was easily cured following treatment. In contrast others thought it was a deadly disease that would kill them after a short duration.

Most only came to understand and accept the chronic nature of diabetes after months or years of experience with the disease and explanations from healthcare providers. An emotional tinge was evident in some of the expressions.

*When told that I had this illness I thought I would die the very next day. I didn’t think that I would get to live for all these years because I had no knowledge about the disease. (Male, 45* *years, high school, 22* *years with diabetes)*

*This illness doesn’t go away once you get it; it is a for life time. I am experiencing it now. While being treated here in this diabetic center, I had once requested them to release me (as cured) because the levels used to go very low. But they told me that once you get diabetes it doesn’t go away and that one cannot say I don’t want it any more. (Female, 50* *years, diploma graduate, 14* *years with diabetes)*

### Impacts of diabetes

Diabetes was initially considered by some to be a condition that would not lead to serious consequences, but this often changed as the illness duration increased. The participants cited a number of diabetes-related consequences ranging from limited to severe physical and social problems.

*Physical health problems*: Some of the physical health problems cited include liver, kidney or heart complications, loss of eye sight, leg amputation, and paralysis of parts or the whole of the body that could also lead to loss of speech were mentioned. Most of these were considered to eventually lead one to become home-bound or even bed-ridden and thus a burden to family members and relatives. Expressions were filled with emotional terms such as being fearful, troubled, and hateful when discussing about these problems especially as they related to being a burden to family.

*My father died after being paralyzed. He was not able to speak. I hate not being able to talk; I also hate losing my sight. It could completely blind you. I fear that a lot. (Male, 45* *years, high school, 2* *years with diabetes)*

*I worry a lot as to whether I die now or tomorrow. I wouldn’t wish diabetes for anyone. I worry quite a lot if it would kill me today or tomorrow…. Diabetes usually takes you while sleeping, no will or anything like that. It could also make you fall while traveling. I don’t go out without having my wife by my side. (Male, 55* *years, low education, 25* *years with diabetes)*

Diabetes was further thought to be an uncontrollable illness by some who considered that severe complications or debilitating sugar level fluctuations occur despite taking precautions or without any warning in turn leading to severe disability or even death. Participants’ expressions had emotional components mostly related to fear.

*The sugar may go up or down and go out of control which may lead to one becoming bed*-*ridden. This could cause disturbance in the family and is what I fear. You are not dead or you won’t get well*—*this problem makes one fearful indeed. (Male, 50* *years, diploma graduate, 7* *years with diabetes)*

*I fear that my sugar level suddenly increases and kills me. There is also hypertension and it could be troublesome. Many people who followed treatment with us have died this year alone as a result of sudden increase in their sugar levels. (Male, 63* *years, high school, 3* *years with diabetes)*

*Social consequences*: Diabetes was also seen to affect social interactions by making some to become short-tempered and in a few other cases affecting relationships with spouses secondary to physical consequences such as impotency, which was a problem especially among the younger male participants. Other physical diabetic-related problems described as having social and economic consequences included diminished eyesight and body strength, since these were considered essential for fulfilling daily activities such as simple household chores, other communal chores or going out to fulfill religious duties. Problems related to limited mobility and eyesight have led to an early retirement for some due to their inability to carry out duties at their former workplace.

*In fact the disease makes you ill*-*tempered; you get irritated even for minor things. (Male, 50* *years, diploma graduate, 7* *years with diabetes)*

*My eye has also created problems for me. It is only when I bake injera [a flat spongy bread made from teff] that my eyes black out and so I no more bake injera. In my neighborhood I participate in idir [neighborhood/community association that provides support to households in times of death and funerals]. But because of my eye problem, to avoid dust and smoke I don’t participate in that often. My body and my strength is not right. I do go out for example to the church. The maid does all the household chores and I help with some of the food and so it is like this. (Female, 58* *years, low education, 8* *years with diabetes)*

There were also reports by a few of the participants in relation to social stigma which had implications on social life and possibly on diabetes care.

*At my place of work, there was an individual who didn’t tell us about his illness as if it were something wicked and we heard about his admission to a hospital. Even he did not reveal about his illness to his wife. Anyway, his new shoe led to toe amputation and his leg after that. After that he lost hope, got weak and then it was all over. (Female, 55* *years, diploma graduate, 9* *years with diabetes)*

*That time I had fear going out of the house as my weight was decreasing and fear of people talking about me. I myself had heard one individual telling his friend about me having “drawn” the joker (contracting AIDS). Thereafter I stopped mixing with people. (Male, 45* *years, high school graduate, 22* *years with diabetes)*

### Causation

Many participants expressed uncertainty about the cause of diabetes, but when causes were discussed, heredity and emotional causes were the most commonly expressed while dietary/lifestyle and attribution to the evil spirit/Satan attacks were less common ones mentioned.

*I really don’t know! I can’t tell how I got it. Of course I have hereditary inheritance; almost all my family members are on insulin. (Male, 45* *years, high school, 2* *years with diabetes).*

Negative emotions such as anger, rage and being upset related mostly to social interactions were the most common and widely mentioned as a sure cause for diabetes. Grieving for an extended time and being startled were also mentioned as probable causes believed to have immediately led to the diabetes condition. Such emotions were not only considered to be causes but also as exacerbating factors for those living with diabetes.

*The cause for my diabetes and that of others may be different. As for me I had cattle and one day I had 2000 birr prepared in order to buy frushka (a semi*-*processed cattle feed). When a merchant who went around selling dried grass (another animal feed) came to the house, I put the money away and went out to buy that instead. When I came back for the money, it was nowhere to be found. I turned mad and was so enraged and didn’t know where to go. People rebuked me for that but I was so furious and couldn’t let go easily. It is my guess that is the cause of my diabetes. In fact my children now talk about my actions then that led to this illness and so that is what I and my children now believe as cause of my illness. (Female, 73* *years, low education, 20* *years with diabetes).*

*It had gone down but last time I was in mourning and because of that reason it went back again. It doesn’t like mourning. (Female, 59* *years, elementary school, 9* *years with diabetes).*

### Treatment

Biomedical treatment, religious healing and traditional medicines were described as being useful for the treatment of diabetes—be it control or cure.

Diabetes was believed by many participants to be a controllable illness if the biomedical treatment regimen is followed.

*If one takes good care of it, one can live peacefully for many years… I think that diabetes is better compared to other diseases; the main thing is to take care of oneself. Had it not been for one’s own negligence, it would be possible to control it. (Male, 56* *years, bachelor’s degree, 15* *years with diabetes).*

There were a few however, who perceived that the biomedical regimen could lead to cure from diabetes if the recommended regimens such as diet and medications are followed. Some would express cure via following the biomedical regimen while adding the importance of God’s will for the success of the biomedical regimen to effect cure.

*Yes I think that I will be cured. I believe that God will cure me. By improving my food, God will support me when I act. My preparation is needed and so I have great hope of being cured by correcting my food, exercising and taking my medicines properly. (Female, 48* *years, low education, 18* *years with diabetes).*

Religious healing practices, especially holy water and prayers have been cited by Christians for their benefits in managing diabetes complementary to the biomedical regimen and also popularly described to cure diabetes. Muslims participants apparently did not subscribe to any healing practices other than praying which was described as not specific to diabetes. Holy water was the most popular among the majority Orthodox Christian participants, with prayers cited more commonly among Protestant Christians.

*If I drink lots of it [holy water] continuously it has benefits; it decreases it (sugar levels) by 35, 40. (Female, 33* *years, high school, 6* *years with diabetes).*

*Some people claim that holy water cures. There are even some who promote for people to go there. Now my friend’s wife has been cured and examinations have confirmed it. (Male, 63* *years, high school, 3* *years with diabetes).*

Traditional medicines, specifically medicinal plants were primarily mentioned for their benefits in controlling sugar levels. Among these *shiferaw* (*Moringa* spp), was described as the most popular for diabetes. Others mentioned included *anamuro* (*Ajuga* spp) and kosso areqi (traditionally made hard liquor prepared from *Hagenia abyssinica*).

Scientific breakthroughs were another hope for a cure that was cited. In this regard, ‘recent’ media reports that have apparently described scientific research about a cure for diabetes and that are ‘nearing fruition’ were mentioned. A lifestyle free from negative emotions such as enragement and mourning or where they are controlled were also considered as one of the ways to help mitigate the illness.

## Discussion

This study is the first to report an in-depth exploration into the explanatory models of Ethiopian patients’ with diabetes mellitus. The analysis of the explanatory models focused on a number of issues including the meanings these study participants attach to the label, the symptoms, causation, course of illness, social and physical impacts of illness and treatment as well as their associated negative emotions. It was apparent from the findings that participants had explanatory models that were a mix of the biomedical model and traditional models, with the latter reflecting strong cultural and religious influences. Even though the findings cannot be generalized, the explanatory models identified in this study will offer greater insight to the illness perceptions and experiences of Ethiopian patients with diabetes attending healthcare in similar contexts.

According to the findings, symptoms played important roles in patients’ decisions about their ill health status and care-seeking although most did not identify the symptoms as related to diabetes and were informed of their diagnosis at the health facilities. Low knowledge about the symptoms, or for that matter about diabetes in general, and lack of discernible symptoms have also been identified in studies as a reason for the lack of early detection and the delays in healthcare seeking [14, 15].

With regards to illness causation, many expressed uncertainty while those who were certain primarily cited emotional causes or heredity for their diabetes. The uncertainty about the cause could be influenced among other things by the low public awareness about diabetes as has also been reported in other similar settings [16]. Most of the causes cited in the present study including the rage in relation to social interactions were either external factors or beyond individuals’ control which could have negative implications for their decisions about adherence to recommended regimens and with potential to try out other healing practices such as religious healing and traditional medicine which are thought to be better suited to address illnesses in their cultural and religious contexts [17]. Other studies have reported that when the disease is perceived to have an internal cause, individuals take responsibility and have been associated with better adherence to treatment regimens [18, 19].

There were conflicting ideas with some believing or hoping that diabetes is an acute, curable condition and others maintaining that it was a chronic, controllable condition. It was however interesting to note that the regimen described for both the cure and control of diabetes were mostly similar and included the use of biomedical treatments, religious healing and traditional medicine. One reason for practitioners to understand if their patients were expecting a cure is that some may be carefully following the biomedical regime in hope of a cure which may over time lead to frustration if their goal is not fulfilled. This is in turn could lead to lowering adherence to the recommended regimen or trying out different healing practices such as religious healing or traditional medicines which would have the potential to adversely affect the treatment regimen one way or another and affect health outcomes. Reports from other studies done in similar settings report how hope for a cure could actually lead patients try out traditional medicines which is a culturally appropriate approach, albeit with suboptimal health outcomes [20, 21].

Religious healing and traditional medicine were control approaches for diabetes that could be used on a complementary or sometimes alternative to the biomedical regimen in the present study. The use of religious healing such as holy water which is thought to cure diabetes, control sugar levels or otherwise manage related complications may prompt patients to reduce or even discontinue their medicines to observe the effects. On the other hand, use of traditional medicinal plants alongside the biomedical regime may lead to interactions with the possibility of lowering sugar levels and hypoglycemia if the herb indeed has hypoglycemic activity as has been reported for *shiferaw* (Moringa) [22]. It could also lead to discontinuation of the biomedical treatment in favor of traditional medicine, whose safety and efficacy have not been proved. Shifting to religious or traditional medicines were in fact among common reasons cited by psychiatric patients for not adhering to their medications according to a study reported from a hospital-based study in the southwestern part of the country [23]. Going to holy water sites to be baptized was also among the frequently cited reasons for HIV patients being lost to follow up according to a study reported from the northwestern part of Ethiopia. Measures recommended by some priests of Orthodox Christianity to be cured of HIV/AIDS included taking holy water, praying to God and stopping medication taking [24]. Efforts to mitigate some of these challenges include the collaborations forged between biomedical practitioners and the Ethiopian Orthodox Tewahedo Church where encouraging results with regards to improved adherence were reported in the treatment of mental illness and HIV/AIDS [25, 26]. The perceptions of priests involved in the religious healing of these patients and possibility of collaborations could be one of the areas to be explored for the management of diabetes as the combined use of these treatments could also benefit spiritual and mental health, which are not well-addressed by the biomedical system [25].

A number of the study participants also portrayed diabetes as a deadly disease or as an uncontrollable condition which would inevitably lead to severe physical and social consequences despite following treatment recommendations. It is interesting to note that the poor control and high rate of complications widely described by our participants has also been reported by an Israeli study done among immigrants of Ethiopian origin there. The author surmised that the migrants have little experience of ‘living with and controlling a chronic illness’ and that management was not a common term in relation to disease [27]. While negative emotions are not unexpected, such strong emotional expressions which depict diabetes as a deadly disease, the fatalistic nature of the diabetes-related complications and its uncontrollable nature could potentially prove destructive for these participants’ adherence to recommended treatment regimen and their health outcomes [6, 28, 29]. Negative emotions could also strengthen tendencies to try out healing systems promising cure or better control and further result in poor patient outcomes [30, 31].

The negative emotions that have been identified by participants of this study as possible causes for diabetes as well as the emotional distress reported by some study participants suggests the need for consideration of the influence of emotions in the patients’ illness experience and its management. This is supported in the literature where others have identified a possible relationship between diabetes-related distress and depression with type 2 diabetes, whereby the diabetes is associated with an increased risk of incident depression and vice versa [32, 33]. While it is possible that depression may coexist in some of the study participants as has also been reported in another local study [8], the existence of unique diabetes-related distress seems to be evident as well. Depression and diabetes-related distress in turn have been related to suboptimal adherence levels and poor glycemic control [32, 33]. This may hint of the possible benefit that patients with diabetes may get with regards to their illness should some form of mental health treatment such as psychotherapy be included for their distress and depression, if present. As there is currently no mental health service in the routine diabetes care, it is recommended that diabetes management should consider its incorporation in the consultations, be it individual patient-centered or group education sessions, in addition to the context-related diabetes educations in order to improve patient outcomes with regards to mood but also those that are diabetes-related [32, 33].

### Practice and research implications

The findings of this study have highlighted important issues for consideration in the care of patients with type 2 diabetes. One of these is study participants’ perceptions that are strongly informed by religious and cultural influences and their low awareness about diabetes and its biomedical management. The other issue is the emotional distress that seems to exist among some of the study participants. These issues signify the need for a rigorous group diabetes education program that addresses local concerns and scenarios such as the ones identified in this study. Diabetes health education topics may include, but are not limited to, the role and implications of symptoms and other monitoring mechanisms such as blood glucose measurements, the chronic and controllable nature of diabetes and evidence-based approaches to management and expected outcomes, as well as causes. Incorporating mental health components, be it in the form of psychotherapy or others appropriate would be useful additions on a need basis. An important issue to consider is the use of innovative training programs such as group sessions that could entail active participation of patients both in the discussions and with regards to their responsibility in their care [34]. The use of diabetes patients who have good control of diabetes can also be considered to share their experiences and further serve as models about the controllable nature of diabetes.

The diabetes education program discussed above should complement but not substitute one-to-one consultation of patients with healthcare providers. It was apparent from the study that participants differed in their levels of biomedical knowledge and religious and cultural influences and as well with the levels of emotional distress expressed which would be indicative of the different management and education needs of these patients. With regards to the emotional issues, it is recommended that providers regularly assess and monitor patients for depression [33]. This would indicate the need for a more tailored and patient-centered approach to education that addresses patients’ specific needs in understanding and managing their diabetes. Healthcare providers may evidently need additional trainings on different areas including health communication [9], fundamentals of identification and management of depression [33] and as well about common traditional and religious healing practices if they are to effectively care for their patients and offer meaningful advice.

Further research is recommended to assess the explanatory models on a large population sample and design and assess specific interventions that include patient education and mental health treatment components aimed at improving adherence to recommended regimens and consequently health outcomes. Further ethno-pharmacological research is also recommended into the most common traditionally used medicinal plants and so that the findings can be used to provide evidence-based recommendations to patients. Studies to assess the explanatory models among individuals without diabetes in the Ethiopian and other low and middle income settings where diabetes is growing as a public health problem could also serve as useful inputs in the design of health education programs for the prevention and early detection of type 2 diabetes as well as its appropriate care.

## Limitations

Among the limitations of this study is that study participants were recruited from among those following their biomedical treatment in hospitals and those that were not on follow up were not included. The findings expectedly would not represent those who may have avoided the biomedical option and instead chosen traditional, religious or other options. The hospitals where these participants were recruited were public hospitals which serve the relatively lower socio-economic group of population and these perceptions may not be applicable to those who would go to private health facilities and the relatively better-off. Furthermore, all study participants had had diabetes for at least a year when they were recruited and thus the perspectives of newly diagnosed patients would not be included. Finally, this study was limited to exploring the perceptions of patients and did not involve the perspectives of healthcare providers as well as the religious healers caring for these patients.

## Conclusions

The findings of this study indicate that study participants’ explanatory models were a mix of traditional and biomedical models to which emotional distress was an additional component. This model was useful in identifying the different issues that may influence patients’ care for their diabetes which could affect their adherence to recommended regimens and health outcomes. It is therefore recommended that diabetes education sessions, both group and one to one, need to be strengthened including the incorporation of some form of mental health components and be delivered in a manner that consider local contexts and be patient-centered.

## Appendix

### Interview guide

1. When did you find out you had diabetes? How did you feel when you were told you had it? Why do you think your diabetes started when it did?
2. What do you think has caused your diabetes?
  - Can you think of anything else that may cause diabetes?
3. What are the symptoms of your diabetes? Have you encountered any stigma or discrimination because of your diabetes or some of its symptoms?
4. What do you think your diabetes does to you?
  - How does it work?
  - What do you fear most about your sickness?
5. Will your illness have a short or long course?
  - How long does it take to get over this illness?
6. What are the chief problems this illness causes for a person?
  - What are some obstacles to your day-to-day management of diabetes?
7. How severe is your sickness? Does diabetes get better or worse the longer you have it?
8. What can people do to take care of this illness? What works and what doesn’t?
